# Structural brain morphometry as classifier and predictor of ADHD and reward-related comorbidities

**DOI:** 10.3389/fpsyt.2022.869627

**Published:** 2022-09-12

**Authors:** Daan van Rooij, Yanli Zhang-James, Jan Buitelaar, Stephen V. Faraone, Andreas Reif, Oliver Grimm

**Affiliations:** ^1^Department of Cognitive Neuroscience, Donders Institute for Brain, Cognition and Behavior, Radboud University Medical Center, Nijmegen, Netherlands; ^2^Department of Psychiatry and Behavioral Sciences, SUNY Upstate Medical University, Syracuse, NY, United States; ^3^Department of Psychiatry, Psychosomatic Medicine and Psychotherapy, University Hospital, Goethe University, Frankfurt, Germany

**Keywords:** ADHD, comorbidity, machine learning (ML), morphometry, SUD, depression

## Abstract

Attention deficit/hyperactivity disorder (ADHD) is one of the most common neurodevelopmental disorders, and around two-thirds of affected children report persisting problems in adulthood. This negative trajectory is associated with high comorbidity with disorders like obesity, depression, or substance use disorder (SUD). Decreases in cortical volume and thickness have also been reported in depression, SUD, and obesity, but it is unclear whether structural brain alterations represent unique disorder-specific profiles. A transdiagnostic exploration of ADHD and typical comorbid disorders could help to understand whether specific morphometric brain changes are due to ADHD or, alternatively, to the comorbid disorders. In the current study, we studied the brain morphometry of 136 subjects with ADHD with and without comorbid depression, SUD, and obesity to test whether there are unique or common brain alterations. We employed a machine-learning-algorithm trained to classify subjects with ADHD in the large ENIGMA-ADHD dataset and used it to predict the diagnostic status of subjects with ADHD and/or comorbidities. The parcellation analysis demonstrated decreased cortical thickness in medial prefrontal areas that was associated with presence of any comorbidity. However, these results did not survive correction for multiple comparisons. Similarly, the machine learning analysis indicated that the predictive algorithm grouped most of our ADHD participants as belonging to the ADHD-group, but no systematic differences between comorbidity status came up. In sum, neither a classical comparison of segmented structural brain metrics nor an ML model based on the ADHD ENIGMA data differentiate between ADHD with and without comorbidities. As the ML model is based in part on adolescent brains, this might indicate that comorbid disorders and their brain changes are not captured by the ML model because it represents a different developmental brain trajectory.

## Introduction

Attention deficit/hyperactivity disorder (ADHD) is one of the most common neurodevelopmental disorders and has prevalence in children of 5–7%, of which around two-thirds report persisting problems in adulthood (1, 2). A major factor contributing to the disease burden of ADHD in adolescent and adult populations is the high rate of comorbidities in ADHD (3). Across the lifespan, increased prevalence of affective disorders, personality disorders, and substance use disorder (SUD) is present in patients with ADHD as compared to the general population (4–6). Aside from reporting a higher disease burden (7), subjects with ADHD and comorbid disorders also suffer from decreased treatment efficacy (8) and higher lifetime mortality (7). These issues underline the need for more research on underlying mechanisms leading to high comorbidity in subjects with ADHD.

Alterations in brain morphometry have been widely reported in both children and adults with ADHD (9, 10) and have been associated with clinical factors like disease burden, persistence, and outcome of interventions (11, 12). Studied separately, decreases in cortical volume and thickness have also been reported in depression (13), SUD (14, 15), and even obesity (16, 17), but it is unclear whether these structural brain alterations represent unique disease profiles or whether they represent a common underlying mechanism that might link ADHD to these disorders (18). In a review of structural MRI studies, Radonijc et al. (19) reported a significant correlation in volumetric changes between sMRI findings in ADHD and other corresponding mental health disorders like major depression. In the current study, we will study the structural brain morphometry of subjects with ADHD with and without comorbid depression, SUD, and obesity to test whether there are unique or common alterations underlying each of the comorbidities of ADHD. One of the key overlapping features between ADHD and the three abovementioned comorbidities is altered structure and functioning of the reward network in the brain (18). Based on previous findings, we expect potential common underlying alterations in brain morphometry to be concentrated around the thinner dorsal and medial and frontal cortices, as well as in smaller subcortical brain volumes.

We used two distinct analytic approaches to investigate this topic. First, we conducted a standard univariate analysis of FreeSurfer-based volume segmentations of the structural brain scans of subjects with ADHD with and without comorbidities for testing our specific hypotheses. Second, we employed a machine learning (ML) algorithm trained to classify subjects with ADHD in a larger dataset and used it to calculate the ADHD brain risk score [BRS, e.g., (20)] of the current subjects with ADHD with and without comorbidities. We expect that if the brain morphometry changes associated with ADHD and our comorbid disorders act upon the same neurobiological pathway, the predictive algorithm would find more evidence to classify the subjects as “affected” in the group of subjects with ADHD and comorbidities, as opposed to the group with ADHD only. This would result in higher BRS for the subjects with ADHD and comorbidities. Hence, the two types of analyses can be seen as complementary explorative approaches to assess whether morphometric differences are shared or different between ADHD with and without comorbid depression, SUD, and/or obesity.

We address three separate research questions by both types of analyses: 1) is there a general effect of comorbidities on structural brain morphometry and/or predictive accuracies of the ML algorithm? 2) Is there a cumulative effect of comorbidities? 3) Is there a specific effect of each of the comorbidities?

We hypothesized that the subjects with ADHD and comorbid disorders will show smaller subcortical brain volumes and lower cortical thickness, particularly in the frontal-striatal pathways than the patients with ADHD only (18). We hypothesized that the subjects with ADHD and comorbidities would be more likely to be classified as affected by the algorithm as compared to the subjects with ADHD only, and hence get a higher average BRS. We further hypothesized that multiple comorbidities would be associated with larger structural brain changes and increased BRS. Lastly, we hypothesized that the subjects with comorbid depression would show smaller frontal and hippocampal volumes (21), while for the subjects with SUD and obesity we expect smaller frontal and striatal volumes (22, 23).

## Methods

### Participants

The study includes 133 subjects with ADHD with and without the comorbid presence of depression, obesity, or SUD (men = 57, mean age = 27). The recruitment took place at Donders Center for Cognitive Neuroimaging, Nijmegen, Netherlands and Goethe University, Frankfurt am Main, Germany.

Inclusion criteria were: age between 18 and 55 years, previously established diagnosis of ADHD, and compatibility with MRI acquisition. Exclusion criteria were acute mental illness (excluding the mental comorbidities of focus in the current study, namely, depression and SUD), serious acute or chronic physical diseases, pregnancy, as well as exclusion criteria of the MRI examination. Only patients with at least 4 weeks of stable medication regimen were included. Stimulants, alcohol, and nicotine were stopped on the day of the scan. Patients taking an antipsychotic medication were excluded. Participants were examined by a registered psychiatrist in Frankfurt or a trained research psychologist in Nijmegen. Childhood symptoms in the German ADHD sample were additionally measured *via* the short Wender-Utah-Rating scale [WURS; (24)]. Adult ADHD symptoms were counted with the Diagnostic Interview for ADHD in Adults 2.0 (25), which uses the 18 DSM-5 criteria for ADHD. Participant details can be found in Table 1.

**Table 1:** Participant characteristics.

	**ADHD**	**ADHD with comorbid disorders**
Number of participants *(N)*	32	101
Age (years)	mean = 27,72 (min = 18, max = 43)	mean = 33,84 (min = 18, max = 55)
Site (N)		
Nijmegen	5	56
Frankfurt am main	27	45
Sex (N)		
Female	14	62
Male	18	39
**Number of comorbidities** *(N)*		
1		42
2		50
3		9
**Type of comorbidities** *(N)*		
Obesity		64
Depression		71
SUD		34
**Overlap of comorbidities** *(N)*		
Depression		17
Obesity		19
SUD		9
Depression + Obesity		4
Depression + SUD		13
Obesity + SUD		32
Depression + Obesity + SUD		8
**Medication use** *(N)*		
Stimulants (i.e. Ritalin, Concerta)	7	23
Atomoxetine		2
Antidepressants		2
Other		3

The project was carried out in accordance with the provisions of the Declaration of Helsinki and the European Guidelines on Good Clinical Practice, and was approved by the Ethics Committee of the Medical Faculty of the J.W. Goethe University Frankfurt am Main (reg. no. 256/16) and in Nijmegen by Radboud University (reg. no. 2018-4364).

### Data acquisition

Structural T1 MPRAGE sequences were acquired on a Siemens PrismaFit scanner (Nijmegen) or a Siemens Trio Syngo scanner (Frankfurt). T1 scans were segmented by FreeSurfer cortical and subcortical segmentation. Quality Control (QC) was performed using the standardized ENIGMA QC pipeline (http://enigma.ini.usc.edu/protocols/imaging-protocols/) based on automatic outlier detection and visual inspection of segmentation quality.

### FreeSurfer segmentation methods

In order to investigate brain morphometry, all structural MRI scans were segmented into subcortical volumes, cortical thickness, and cortical surface area by FreeSurfer segmentation. Structural T1-weighted MRI scans acquired at the two contributing sites were segmented using standardized and publicly available ENIGMA imaging protocols (http://enigma.ini.usc.edu/protocols/imaging-protocols/). The automated protocols, based on FreeSurfer (version 5.3) segmentations, are fully validated and allow for maximal uniformity and comparability across sites. For each participant, left and right subcortical volumes, cortical thickness, and cortical surface area measures were calculated. Quality control depended on the visual inspection of all volume segmentations. Poorly segmented regions were removed from further analyses.

### Machine learning training sample

We used an existing machine learning algorithm that was previously trained and validated in the very large ENIGMA-ADHD data cohort to classify subjects with an ADHD diagnosis from healthy controls on the basis of structural brain data. This previous study found the predictive accuracy, as measured by the area under the receiver operating curve, to be.62, indicating it is significantly better than chance prediction of ADHD or control status (CI = 0.56–0.69, *p* = 0.002). Full details of the model training and operation are available in Zhang-James et al. (26). The code for generation of the ML model can be found on https://github.com/ylzhang29/ADHD_MLP and access to the ENIGMA consortium can be requested through https://enigma.ini.usc.edu/about-2/.

The ENIGMA-ADHD training data were based on T1-weighted structural MRI (sMRI) data from 4,183 subjects (adults and children with and without ADHD) from 35 participating sites (by Aug. 2019, see 9; 26). Images were processed using the consortium's standard segmentation algorithms in FreeSurfer (V5.1 and V5.3). Details about the processing of the training data sample are provided in Zhang-James et al. (27). FreeSurfer standard segmentation was conducted as described in the ENIGMA protocol. Final sMRI features used in the classification modeling were 151 variables including 34 cortical surface areas, 34 cortical thickness measurements, and 7 subcortical regions from each hemisphere, and intracranial volume (ICV).

For the current study, the algorithm was applied to our new comorbidity cohort, rendering a BRS for each subject, indicating the likelihood that the subject was affected by ADHD. Average BRS scores were used in the current study to test whether predictive accuracies for the ADHD diagnostic status would differ between subjects with ADHD and comorbid diagnoses of SUD, obesity, or depression.

### Machine learning feature preprocessing, algorithms, and model evaluation

**Figure 1:**
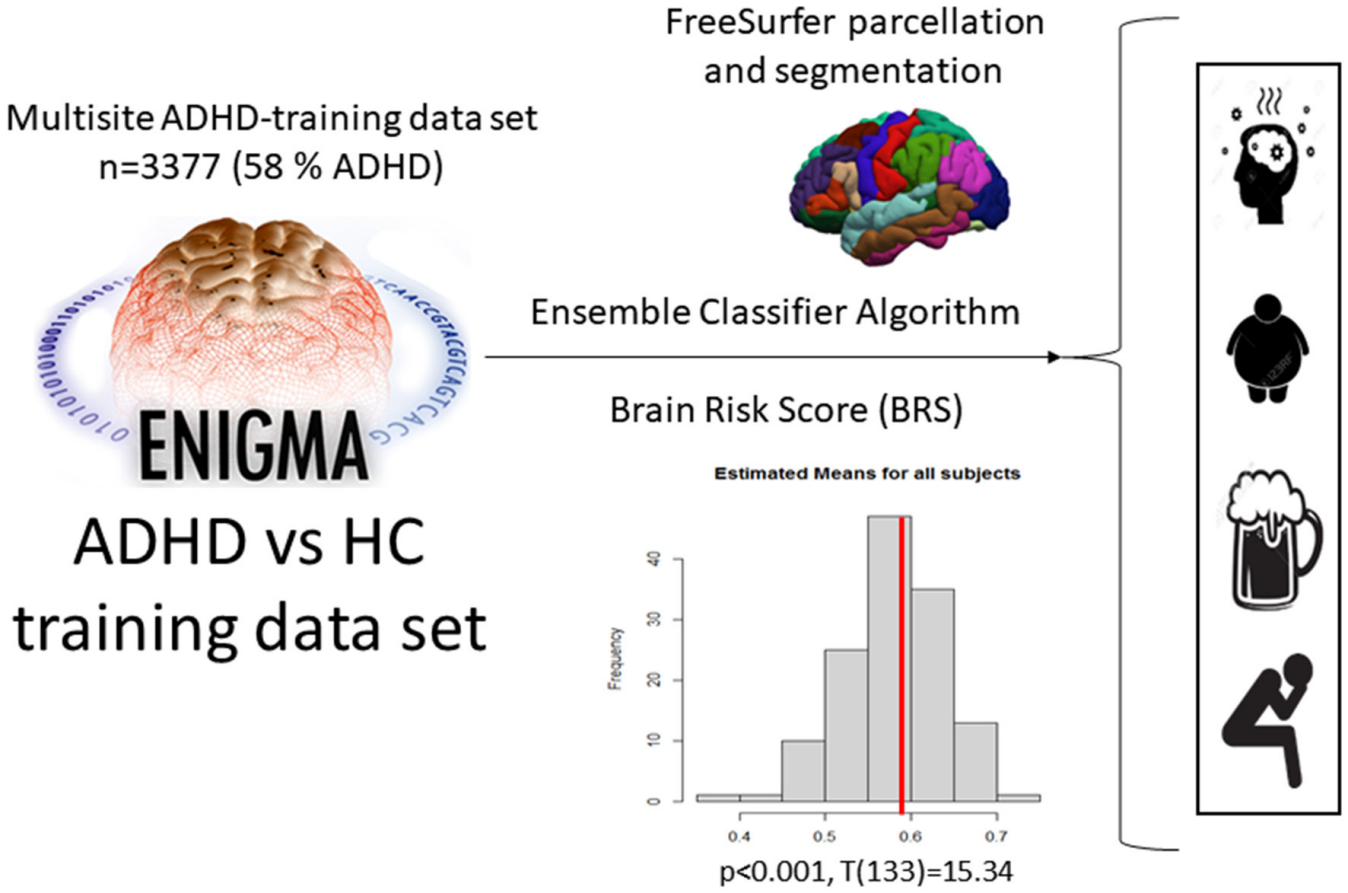
Application of the ML ensemble classifier algorithm, which was used to estimate the BRS for ADHD in our current sample based on the ENIGMA cohort data, and then applied to classify different comorbidities in ADHD.

The machine learning classifier reported by Zhang-James et al. (26) was used to calculate a risk score for ADHD based on the aforementioned subcortical and cortical thickness and surface area segmentations to study differences between comorbidity subgroups of patients with ADHD (Figure 1). As a first data reduction step before the training of the classifier, principal factors factor analysis (PFFA) was performed with varimax rotation on all the 151sMRI features in the original ENIGMA-ADHD training set, and identified 46 factors that explained > 90% of the total variance in the training set. The original study compared the performance of the 46 non-correlated factors with the original 151 sMRI features and determined that models that used the PFFA factors as input features achieved better performance than the original sMRI features. Therefore, in the current study, we computed the 46-factor scores for all the subjects in our comorbidity cohort based on the PFFA from the ENIGMA training set. The factor scores were also scaled based on the training set's minimum and maximum values before the prediction algorithm was used, as described in Zhang-James et al. (26).

The final ensemble multilayer perceptron (MLP) neural network models reported in Zhang-James et al. (26) were tested on the input PFFA features from our comorbidity cohort. Specifically, the model was re-trained on the original ENIGMA-ADHD training and validation sets and tested on our comorbidity cohort. The MLP model uses a sigmoid function in the final layer to generate a continuous brain risk score (BRS), which assesses the probability for each individual to be diagnosed with ADHD. The final predicted BRS was based on a bootstrap averaging (bagging) ensemble of MLP models (26).

The primary outcome measure in the current study was therefore the BRS for each subject, indicating a likelihood that each subject would be classified as affected by ADHD. Given that all our subjects have an ADHD diagnosis (both in the comorbid and non-comorbid groups), correct prediction would mean the algorithm classifies all the subjects as “affected”.

### Statistical analyses

As stated in the introduction, we formulated three hypotheses to test for the effects of comorbidity on structural brain morphometry, as operationalized by FreeSurfer segmentations of subcortical volumes, cortical thickness, and cortical surface area. First, we compared patients with and without comorbidity. Second, we looked for a linear effect of comorbidity (none, one, two, or three). Third, we compared by F-test contrast all eight subgroups of different combinations of depression, SUD, or obesity. All tests included age, gender, and site as covariates. Intracranial volume (ICV) was added as an additional covariate in all the models, with subcortical volume as the dependent variable. The first two tests were defined as *t*-tests and the third one as F-test.

We calculated differences in medication use between the comorbidity subgroups with a univariate general linear model in R (R Core 27). Comorbidity (SUD, obesity, and depression) was used as a dependent variable, whereas medication type (stimulant, atomoxetine, antidepressant, none, other) was used as an independent variable with age, sex, and site as covariates.

## Results

### Freesurfer results

In accordance with the three research questions, three sets of models were run in R. Each model used each segmentation of subcortical volume, cortical thickness, and surface area as separate independent variables. The first model set tested for differences between participants with ADHD and no comorbidities and those with any comorbidity. The second model set tested for group differences between participants with ADHD and all comorbidities (cumulatively). The third model set tested for individual differences among subjects with ADHD, ADHD + depression, ADHD + obesity, and ADHD + SUD.

The summarized results of the models are depicted in Table 2 (full results are depicted in Supplementary Tables 1, 2). The analyses show an uncorrected group difference between subjects with solely ADHD and ADHD + any comorbidity in the right putamen (B = −6.933, *p* =0.022), left bank of the superior temporal sulcus (B =0.005, *p* = 0.009), and middle temporal sulcus (B = 0.004, *p* = 0.023). Similarly, the model testing differences between solely ADHD and ADHD + all cumulative comorbidities showed uncorrected group differences in the right and left rostral middle frontal (B = −0.052, *p* = 0.033; B = −0.068, *p* = 0.004 respectively), superior frontal (B = −0.072, *p* = 0.003; B = −0.057, *p* = 0.01), right pars triangularis (B = −0.071, *p* = 0.007), lateral orbitofrontal (B = −0.055, *p* = 0.044) and left pars opercularis (B = −0.051, *p* = 0.046), and caudal middle frontal (B = −0.064, *p* = 0.014), indicating lower cortical thickness in frontal areas associated with more comorbidities. These effects did not survive multiple comparisons corrections with a stringent pFDR-correction.

**Table 2:** FreeSurfer results on cortical thickness and subcortical volumes (only uncorrected significant values are displayed.

	**H1: Any Comorbidity**		**H2: Cumulative comorbidities**		**H3: Specific comorbidity effects**		**H1: Any Comorbidity**		**H2: Cumulative comorbidities**		**H3: Specific comorbidity effects**
**Right hemisphere**	**B**	**p-value**	**B**	**p-value**		**Left hemisphere**	**B**	**p-value**	**B**	**p-value**	
**Frontal**						**Frontal**					
Superior frontal	0.001	0.427	−0.072	**0.003**	ADHD < ADHD+ Obesity	Superior frontal	0.001	0.564	−0.057	**0.010**	ADHD < ADHD+ Obesity
Rostal middle frontal	0.001	0.557	−0.052	**0.033**	ADHD < ADHD+ Obesity	Rostal middle frontal	0.001	0.474	−0.068	**0.004**	
Pars orbitalis	−0.001	0.794	−0.069	0.071	ADHD < ADHD+ Obesity	Pars orbitalis	0.003	0.197	−0.019	0.582	
Pars triangularis	−0.001	0.407	−0.071	**0.007**	ADHD < ADHD+ Obesity	Pars triangularis	0.001	0.437	−0.045	0.084	
Lateral orbitofrontal	0.001	0.588	−0.055	**0.044**		Lateral orbitofrontal	0.003	0.081	−0.033	0.206	
Caudal middle frontal	0.000	0.958	−0.032	0.207		Caudal middle frontal	0.000	0.908	−0.064	**0.014**	
**Cingulate**						**Cingulate**					
Rostal anterior cingulate	0.002	0.498	−0.086	0.031		Rostal anterior cingulate	−0.001	0.546	−0.089	**0.016**	
**Temporal**						**Temporal**					
Temporal pole	0.004	0.264	0.134	**0.028**		Temporal pole	0.000	0.925	−0.091	0.177	
Bankstats	0.003	0.166	−0.024	0.470		Bankstats	0.005	**0.009**	0.014	0.635	
**Striatal**						**Striatal**					
Putamen	5.148	0.468	9.499	0.934	ADHD < ADHD+ Obesity	Putamen	1.417	0.844	16.556	0.887	
Pallidum	−6.933	**0.022**	−40.530	0.421		Pallidum	−1.614	0.569	−56.925	0.210	
Amygdala	0.654	0.799	10.075	0.808		Amygdala	0.300	0.908	13.401	0.750	ADHD < ADHD+ Obesity

Bold values indicate p-values below 0.05 (uncorrected). No observed FDR-corrected p-values were lower than 0.2.

No effects of the different comorbidities on medication use were observed. As an exploratory follow-up analysis, medication use (as an ordinal variable sorted by type: stimulant, atomoxetine, antidepressant, none, other) was also subsequently added as a predictor in all ROIs that showed an uncorrected significant effect as depicted in Table 2. No significant effects of medication use on any brain measure were found.

### Machine learning results

The ML algorithm-predicted BRS of ADHD was, on average, 0.57, indicating that the algorithm, on average, found more evidence that the subjects were diagnosed with ADHD than that they were unaffected (t = 15.58, *p* < 0.001). However, no differences were observed between any of the comorbidity groups, so the addition of comorbidities did not influence the accuracy of this prediction. Neither any, all, or each of the comorbidities were associated to BRS (any: t = 0.445, *p* = 0.656, Cohen's d = 0.18, Cohen's d CI = −0.21–0.59; all (cumulative): t = 0.879, *p* = 0.381, Cohen's d = 0.15, Cohen's d CI = −0.3–0.62; every: f = 0.04, *p* = 0.986). The *post hoc* power analyses indicate that the power achieved for the F-test was 1-β = 0.05, limiting the interpretability of the lack of effects. BRS was not associated with site or sex, and neither was the BRS correlated with childhood symptoms of ADHD (WURS-k) (refer to Supplementary Figures).

## Discussion

Our segmentation analyses show some small effects of comorbidities on decreased cortical thickness in the frontal areas of the brain, but none of these effects survive the correction for multiple comparisons due to the large number of segmentations tested.

We found nominally significant reductions in the cortical thickness of several parts of the frontal lobe especially in the rostral middle gyrus in patients with a higher number of comorbid disorders independent of specific disorder type. This points to mechanisms that may be related to a general psychopathology (p)-factor (28) in the disease process leading to a pattern of common underlying structural brain changes associated with presence of mental disorders, which could become more pronounced in the presence of multiple comorbid disorders. In fact, a recent review article (18) reports a finding of decreased frontal cortical thickness in subjects with ADHD and comorbid depression, SUD, or obesity; this is in line with a p-factor model but in contrast to our current findings. Additionally, we observed some nominally significant reductions in frontal cortical thickness mainly in the ADHD + obesity group, suggesting some differentiation between comorbidities in their association with specific structural brain alterations in the superior and medial frontal cortices. These results, however, also did not survive more stringent correction for multiple comparison (FDR correction).

Similarly, the ML algorithm had a predictive accuracy of 0.57, indicating an above-chance level for a correct ADHD diagnosis across all the subjects. However, this did not differentiate between subjects with solely ADHD or subjects with ADHD and comorbidities. Therefore, an ML model trained to differentiate between subjects with ADHD and healthy controls in the ENIGMA-ADHD dataset does not differentiate between subjects with just ADHD and ADHD + comorbidities. This indicates that the features used by this data-driven algorithm to predict ADHD status are not the same features that are altered in subjects with comorbidities. Therefore, using this approach, we find no evidence that the underlying morphometric features underlying ADHD and comorbid disorders are similar.

As the ML model was based at least in part on adolescent brains, this might indicate that comorbid disorders and their brain changes that come later in life are not captured by the ML model because it represents a different developmental trajectory. In addition, one could argue that the disorders are not simple extensions of a dysregulated ADHD-developmental trajectory; therefore, our ML model does not pick up different subgroups.

Several further limitations should be considered when discussing our study. First, the limitations of the ML model described by Zhang-James et al. (26) apply to our study. The ENIGMA mega-analysis combined heterogeneous data from many sites. Finally, there was a gender difference in children and adolescents. Zhang-James et al. (26) therefore discusses whether there is increased noise in the combined dataset that makes it difficult to classify patients with ADHD. Furthermore, our study only considers structural morphometrics. Data such as resting-state connectivity or DTI based connectivity metrics could be a promising alternative. In contrast to our univariate analyses of FreeSurfer-segmented brain regions, an individual risk score cannot elucidate the specific contribution of individual brain regions. A general drawback in our study is the lack of control group, i.e., can our analyses and models differentiate between subjects with ADHD and subjects without? This has been evaluated in detail in Zhang-James et al. (26) using the ENIGMA-ADHD cohort consisting of subjects across the lifespan and indicating that the ML model does indeed significantly differentiate subjects with ADHD from controls. These results were obtained in an average younger sample than the current study. If the ML model captures some ADHD-related developmental aspect, it might be more strongly correlated with childhood symptoms. However, our analysis did not indicate that symptom load in childhood (as measured by the WURS-k questionnaire) was correlated with the model's estimation. Furthermore, our study did not evaluate systematically different ML-models like deep neural networks vs. Gaussian processes. A different ML model might have detected differences between ADHD comorbidities. However, the goal of the current study was to specifically evaluate a previously tested model (26), as our dataset is too small to estimate a separate ML model. In line with this limitation comes the fact that we did not make use of a more fine-grained voxel- or vertex-based analysis technique in our ML-model, but we relied on the parcellation scheme of FreeSurfer. As the best strategy in terms of sensitivity and validity is still unclear, we choose to stay with the previously evaluated model (26). Lastly, it should be noted that the sample size of the current study was quite limited to establish differences between several classes of comorbidities. Both the test accuracy of the ML algorithm and the statistical power of the segmentation analyses will benefit from larger sample sizes to detect potential subtle anatomical differences. The *post hoc* power analyses also indicate low to moderate power for the FreeSurfer analyses (1-β = 0.1–0.2). Therefore, a more extensive sample may aid in uncovering subtle severity-related effects on brain structure.

An innovative part of our study is the use of an ML-model for generating a predictive ADHD brain risk score. This has parallels in recent studies trying to use the ENIGMA dataset as a weighting prior for a brain risk summary measure, e.g., in schizophrenia (29) but not in ADHD. Future studies are needed to compare the simple morphometric brain risk measures in ADHD to our ML Gaussian process model, which might be over the higher sensitivity of ML models.

To conclude, it is notable that neither the morphometric ML-model nor the univariate FreeSurfer-Segmentation models were effective in predicting ADHD and comorbid disorders. However, we do not think that the current results should be interpreted as definitive evidence that there are no effects on individuals with ADHD depending on type or number of comorbid disorders. Future studies might further investigate specific tasks, e.g., reward-learning, and look for relationships with ADHD symptoms. For example, there are alternate approaches with specific tasks, e.g., functional connectivity that may prove better. Indeed, a recent article by our group demonstrated that striatal rs-fMRI-connectivity is able to distinguish between the number of comorbidities aka the disorder “load” (18), indicating this modality as a potential target for future analyses.

## Data availability statement

The raw data supporting the conclusions of this article will be made available by the authors, without undue reservation.

## Ethics statement

The studies involving human participants were reviewed and approved by Ethics Committee of the Medical Faculty of the J. W. Goethe University Frankfurt am Main (reg.no. 256/16) and in Nijmegen by the Radboud University (reg.no. 2018-4364). The patients/participants provided their written informed consent to participate in this study.

## Author contributions

DR and OG were involved in data collection, analysis, conceptual development, and writing of the manuscript. AR and JB were invovled in writing the grant application, conceptual development, and proofreading of the mansucript. YZ-J and SF were involved in the development of the machine learning algorithm, statistical analyses, conceptual interpretation, and proofreading of the manuscript. All authors contributed to the article and approved the submitted version.

## Funding

Financial support for this study was received from the European Union's Horizon 2020 Research and Innovation Programme under grant agreement 667302 (Comorbid Conditions of ADHD).

## Conflict of interest

AR has received a research grant from Medice and served on advisory board and/or speaker's bureau for Medice, Shire/Takeda, Janssen, Servier, and Neuraxpharm. OG has served on advisory board and/or speaker's bureau for Medice Arzneimittel Pütter GmbH and Shire/Takeda and has served on advisory board and/or speaker's bureau for Medice Arzneimittel Pütter GmbH. JB declares that he has been, for the past 3 years, a consultant /member of the advisory board of/and/or speaker for Janssen Cilag BV, Eli Lilly, Medice, Shire, Roche, and Servier. The remaining authors declare that the research was conducted in the absence of any commercial or financial relationships that could be construed as a potential conflict of interest.

## Publisher's note

All claims expressed in this article are solely those of the authors and do not necessarily represent those of their affiliated organizations, or those of the publisher, the editors and the reviewers. Any product that may be evaluated in this article, or claim that may be made by its manufacturer, is not guaranteed or endorsed by the publisher.

## Abbreviations

ADHD: attention deficit/hyperactivity disorder
SUD: substance use disorder
RCT: randomized controlled trial
ML: machine learning
BRS: brain risk score.
