# Supplementary material for: Structural brain morphometry as classifier and predictor of ADHD and reward-related comorbidities
Source: Front Psychiatry. 2022 Sep 12;13:869627. doi: 10.3389/fpsyt.2022.869627 (PMC9512052; doi:10.3389/fpsyt.2022.869627)
Supplement: Supplementary file 5 [file Data_Sheet_1.docx]

Supplementary Figures for “Structural brain morphometry as classifier and predictor of ADHD and reward-related comorbidities”


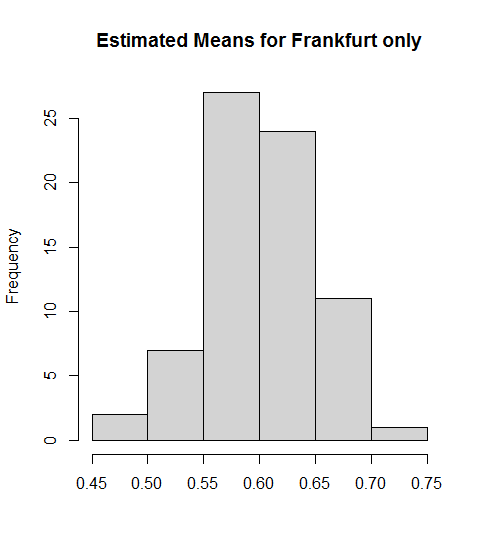


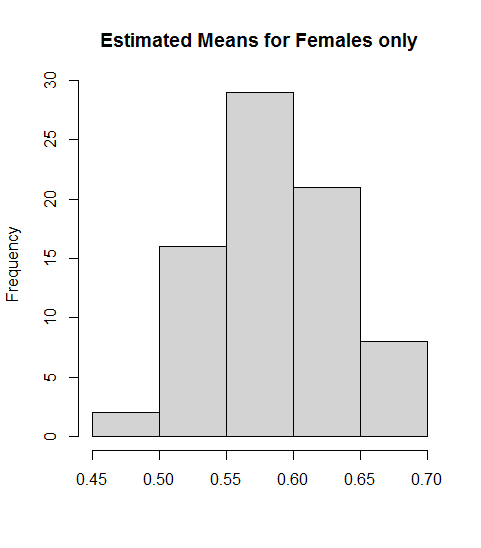

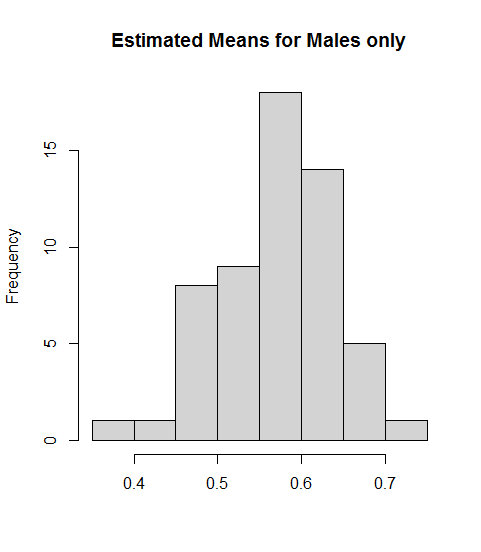

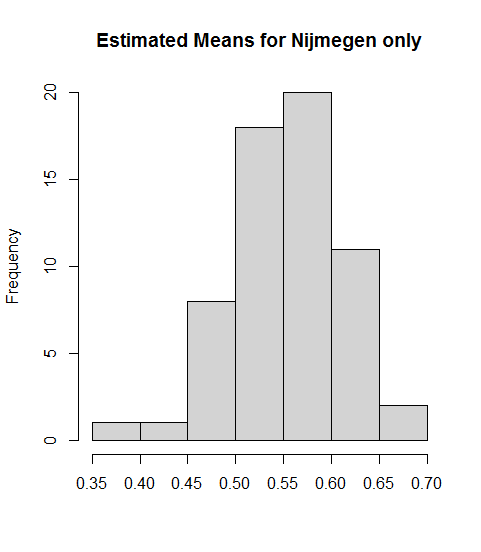

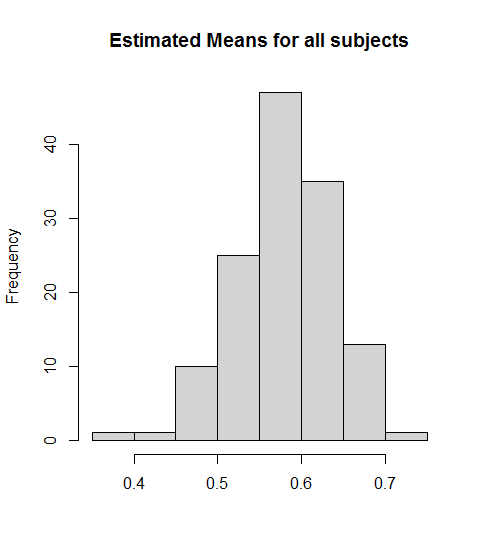


***Supplementary Figure 1****: Histograms showing distribution of Brain Risk Score (BRS) for all subjects, split by site and sex.*


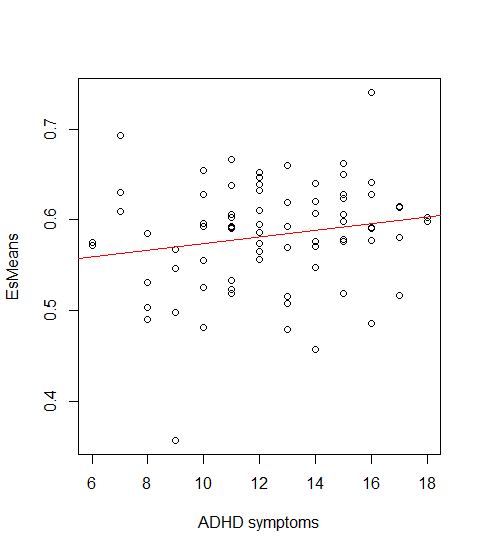

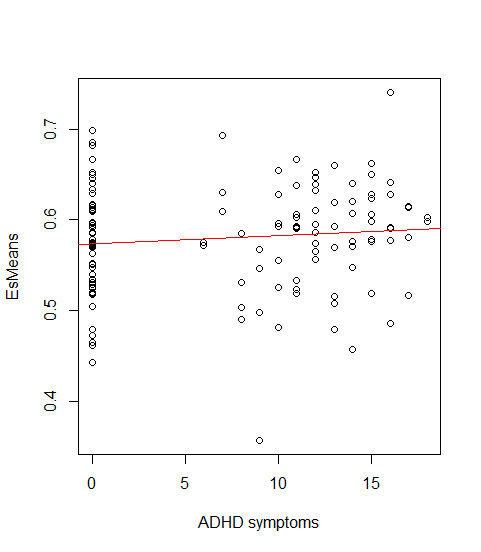


***Supplementary Figure 2: A****. Correlation Brain Risk Score with ADHD symptoms: R=0.10, p=0.23.* ***B*** *Correlation Brain Risk Score with ADHD symptoms above 0: R=0.18, p=0.11*
